# Disinfection Treatments of Disposable Respirators Influencing the Bactericidal/Bacteria Removal Efficiency, Filtration Performance, and Structural Integrity

**DOI:** 10.3390/polym13010045

**Published:** 2020-12-24

**Authors:** Seojin Jung, Tahmineh Hemmatian, Eugene Song, Kyeongeun Lee, Dongwan Seo, Jehyung Yi, Jooyoun Kim

**Affiliations:** 1 Department of Textiles, Merchandising and Fashion Design, Seoul National University, Seoul 08826, Korea; wjdwls04079@snu.ac.kr (S.J.); hanh11@snu.ac.kr (T.H.); songallison@snu.ac.kr (E.S.); kelee@fiti.re.kr (K.L.); dlwpgud2003@snu.ac.kr (J.Y.); 2 Reliability Assessment Center, FITI Testing & Research Institute, Seoul 07791, Korea; sdw5367@fitiglobal.com; 3 Research Institute of Human Ecology, Seoul National University, Seoul 08826, Korea

**Keywords:** respirator, reuse, public health, filtration, electrostatic, mechanical, antimicrobial

## Abstract

In the outbreak of COVID-19, the extended wear of single-use, disposable respirators was inevitable due to limited supplies. As a respirator is front-line protection against particulate matter, including bioaerosol and droplets, a comprehensive understanding for the reuse strategy is needed. In this study, eight different disinfection methods commonly applied for the reuse of respirators were compared for their influence on the filtration and bactericidal/bacteria removal performance, with in-depth discussion on the cause of effects. Treatments including oven-dry, ultraviolet irradiation (UV), microwaving, laundering with and without detergent, and immersion in hypochlorite, isopropanol, and ethanol were performed to respirators. Immersion in ethanol or isopropanol was effective for inactivation and removal of bacteria, yet such a treatment significantly deteriorated the filtration efficiency in about 20–28%, dissipating the surface charges. Laundering, while effective in removing the attached bacteria, triggered physical damage, leading to a possible reduction of filtration performance. A short-term oven-dry, UV irradiation, and microwaving mostly preserved the filtration performance, yet the drawback lied in the incomplete bactericidal efficiency. This study would contribute to the public health and safety by providing scientific background on the effect of disinfection treatment methods for respirators.

## 1. Introduction

The demand for face filtering respirators has grown continuously to cope with various environmental hazards including fine dust, liquid mist, bioaerosol, and droplets. In the meantime, the outbreak of COVID-19 has sculpted a new way of life. Since pathogens can be transmitted via bioaerosol or droplets generated by coughing or sneezing, the use of respirators is now an everyday necessity as the front-line safety tool to protect both the wearer and others from the exposure to such infectious matters [1,2]. According to the Institute of Medicine (IOM), a six-week influenza pandemic results in a demand for 90 million respirators [3]. Likewise, the prevalence of the COVID-19 pandemic has led to a worldwide shortage of respirators, and this situation resulted in the undiscerning reuse of disposable respirators, although most users are not informed of proper methods of respirator maintenance. While little information is available with respect to standardized cleaning methods, commonly used methods to disinfect the used respirators include ultraviolet (UV) irradiation, microwaving, sunlight exposure, laundering, ethanol-spray, heating with a hairdryer, and ironing [4,5]. However, there is a lack of scientific evidence on whether such treatments are indeed effective and safe in disinfecting biological matter and preserving filtration performance [6]. Therefore, it is imperative to investigate the validity and effectiveness of disinfection methods for disposable respirators.

As a contingency strategy for the capacity crisis of disposable respirators, the Centers for Disease Control and Prevention and the National Institute of Occupational Safety and Health announced the guidelines of potential disinfection methods for the reuse of disposable respirators, which include the application of ultraviolet (UV) germicidal irradiation, vaporous hydrogen peroxide (VHP), and moist heat [5]. Table 1 shows the summary of previous studies on respirator disinfection methods [7,8,9,10,11,12,13,14,15,16,17,18,19,20,21,22,23,24]. As for the bactericidal efficiency, autoclaving, UV irradiation, and chemical solvent treatments using bleach, ethanol, and VHP showed up to a 99% reduction of tested bacteria or virus [21,25,26,27,28,29,30]. Nevertheless, some of the results lacked the coherence, and not every method was tested for the bactericidal effect. Thus, a comprehensive investigation is called for to understand the effect of disinfection methods on filtration performance and bactericidal effectiveness.

The particle filtration of an electret media is attributed to the mechanical capture and the electrostatic attraction of particles [31,32]. Many previous studies associated the deteriorated performance of disinfection-treated respirators with the loss of electrostatic charges [7,9,11,15,17,18,19,22,25], yet the direct evidence of charge deterioration was often missing. Additionally, it has hardly been examined for the effect of treatment on the structural integrity of the filter media, which may affect the resistance of respirators. This study aims at divulging the effects of disinfection treatments on inactivation/removal of bacteria, deterioration of filtration performance and structural integrity. To this end, commonly-applied disinfection methods were employed as reuse treatments of respirators, which included microwaving, oven-dry, UV irradiation, immersions in hypochlorite (ClO^−^), ethanol (EtOH), and isopropanol (IPA), and laundering with and without detergent. The influence of disinfection treatment on bactericidal effect was investigated using *Escherichia coli (E. coli)* bacteria, as a common Gram-negative bacteria with viability in diverse environments [33]. The change of filtration performance after treatments and the probable causes for the change were examined by measuring surface potential, wettability, chemical property, and the morphology of filter fibers. The approach of this study is significant in that the validity of various disinfection methods were extensively investigated, associating the deteriorated performance with the physicochemical changes of electret media after treatments. Rarely has it been conducted for this level of inclusive investigation to reveal the impact of disinfection treatments. This study intends to provide practical yet fundamental information on the effect of disinfection treatments in multifarious aspects including bactericidal and filter performance.

## 2. Materials and Methods

### 2.1. Respirators

A commercial respirator certified by N95 grade (coded as Resp. A) and the one certified by the Korean Ministry of Food and Drug Safety (MFDS) for KF94 grade (Resp. B) were used as sample respirators. N95 and KF94 grades refer to the particle capture efficiency of not less than 95% during 200 mg of NaCl particle loading [34] and 94% after 3 min NaCl and paraffin oil loading [35], respectively. The surface area of Resp. A and Resp. B were 249 cm^2^ and 208 cm^2^, respectively.

### 2.2. Disinfection Treatments

#### 2.2.1. Microwave Irradiation

Metal nose clips of respirators were eliminated, and the respirators were exposed to microwave irradiation with 750 W power (MR-280M, LG Electronics, Seoul, Korea); for 1 min, the outer side was directed to the irradiation, then, for 1 min, the inner side was directed to the irradiation. All treated samples were stored for 12 h under ambient temperature before the filtration test.

#### 2.2.2. Oven-Dry

Respirators were dried at 90 °C for 1 h in a drying oven (Withlab Co., Ltd., Gyeonggi-do, Korea).

#### 2.2.3. UV Irradiation

UV rays were radiated from 16.5 cm away from the tray using a UV sterilizer (KRS-A1, KARIS, Gyeonggi-do, Korea). The wavelength of 253.7 nm UV ray was irradiated through the inner area of the sterilizer in 42 cm × 32 cm × 32 cm. The power consumption of UV light bulb was 10 W and the irradiation was conducted for 1 h for inner side and another 1 h for outer side of respirators.

#### 2.2.4. Chlorinated Disinfectant Immersion

Chlorine-based disinfectant (Yuhan Corporation, Seoul, Korea), formulated with sodium hypochlorite (NaClO) (5.5%), sodium hydroxide (NaOH) (0.3%), and water, was used for this treatment. The chlorine disinfectant was diluted in tap water to 5% (*v/v*), which makes the final sodium hypochlorite concentration in water to be 0.275%. The respirator samples were soaked in the solution for 10 min, then rinsed in tap water for 3 min two times, and dried for 24 h under an ambient condition.

#### 2.2.5. Ethanol Immersion

The respirator samples were immersed in an aq. ethanol (EtOH) solution of 70% (*v/v*) for 10 min, then dried for at least 24 h.

#### 2.2.6. Isopropanol Immersion

Isopropanol (IPA) is commonly known as a discharging agent for electret media [36]. Respirators were immersed in IPA liquid (≥99.9%) for 10 min, and dried.

#### 2.2.7. Laundering

Laundering of respirators was done with and without detergent. For water-laundering without detergent, each respirator was put in a stainless can of Terg-O-Tometer (T-O-T, Yasuda Seiki Seisakusho, Tokyo, Japan) with 1 L of tap water. The laundering with water (without detergent) was conducted with agitation speed of 90 rpm at 24 °C for 10 min, and then 3 min for two more times. For detergent-laundering, a detergent (Actz power gel, Pigeon, Seoul, Korea) composed of anionic surfactant was used. Respirators were laundered in T-O-T with 1 L of 0.1 wt% aq. detergent solution at 24 °C and 90 rpm for 10 min, then the samples were rinsed with 1 L of tap water for 3 min four times to thoroughly remove the detergent residue [37].

### 2.3. Electrostatic Force Measurement

The surface potential of filter media was measured using an electrostatic voltmeter (Model 542A, Trek, Lockport, NY, USA), by holding the filter media in the air. A charge-monitoring probe was placed 4 cm above the web surface, and the surface potential was measured by line-scanning over the area.

### 2.4. Filtration Test

The filtration performance of respirators was evaluated using an automated filter tester (TSI 8130, TSI Inc., Shoreview, MN, USA), using NaCl particles (mass median diameter, MMD ~0.6 µm) and paraffin oil aerosol (MMD ~0.4 µm), based on the Korean MFDS standard. NaCl aerosol with a mass concentration of 8 ± 4 mg/m^3^ or paraffin oil aerosol with a mass concentration of 20 ± 5 mg/m^3^ was passed through the respirator sample at the flow rate of 95 LPM. The aerosol penetration after 3 min of challenged aerosol mass and the initial resistance were recorded as performance criteria.

### 2.5. Characterization

For a non-destructive 3D visualizations of an internal structure of materials, X-ray computed tomography (Xµ-CT) was performed using Zeiss X-Radia 510 Versa (Zeiss, Oberkochen, Germany) (Figure S1, see Supplementary Materials) [38]. The X-ray source was operated at a voltage of 60 kV with a power of 5.0 W. The fields of view used were 800, 1200, 2000 µm, and the corresponding pixel sizes were 0.8, 1.2, 2.0 µm, respectively.

FE-SEM images of filter samples were observed by Supra 55 VP (Zeiss, Oberkochen, Germany), with prior coating with Pt at 20 mA for 120 s, using a 108auto sputter coater (Cressington Scientific Inc., Watford, UK). The thickness of respirator layers was measured using a thickness gauge under the pressure of 2.4 N. Porosity and solidity of webs were calculated based on Equations (1) and (2), where m is mass of the material, A is area of the material, t is thickness, and ρ is the material density (0.95 g/cm^3^ for PP was used):

Porosity (%) = (1 − solidity) × 100 (%)(1)

Solidity = m/(A⋅t⋅ρ)(2)

The static contact angle (CA) of liquid was gauged as wetting property using a contact angle analyzer (SmartDrop Lab, FemtoBiomed Inc., Gyeonggi-do, Korea). A droplet of 3.0 ± 0.3 μL liquid, including distilled water (WA), ethanol (EtOH), isopropanol (IPA), chlorinated disinfectant (ClO^−^), and 0.1% detergent solution was dispensed on a surface of web, and CA was measured in 60 s after the droplets were settled. The chemistry of sample surface was analyzed by FTIR-ATR (TENSOR27, Bruker, Germany).

### 2.6. Bactericidal Effect

The *E. coli* strain of KCTC 1039 was used as the test bacteria, cultivated in Luria-Bertani (LB) broth (Sigma-Aldrich, St. Louis, MO, USA) for 2 h at 250 rpm. For application of *E. coli* to respirator layers, 10 μL of bacterial culture, corresponding to ~5 × 10^6^ CFU of *E. coli* (concentration; 5 × 10^8^ CFU/mL), was injected into the center area of 2.5 cm × 2.5 cm of front side of a respirator, using a micropipette. The bacteria-loaded samples were subject to different disinfection treatments, and the CFUs of cells were quantified, using a staining method [39,40]. The quantification procedure is illustrated in Figure S2.

## 3. Results and Discussion

**Figure 1:**
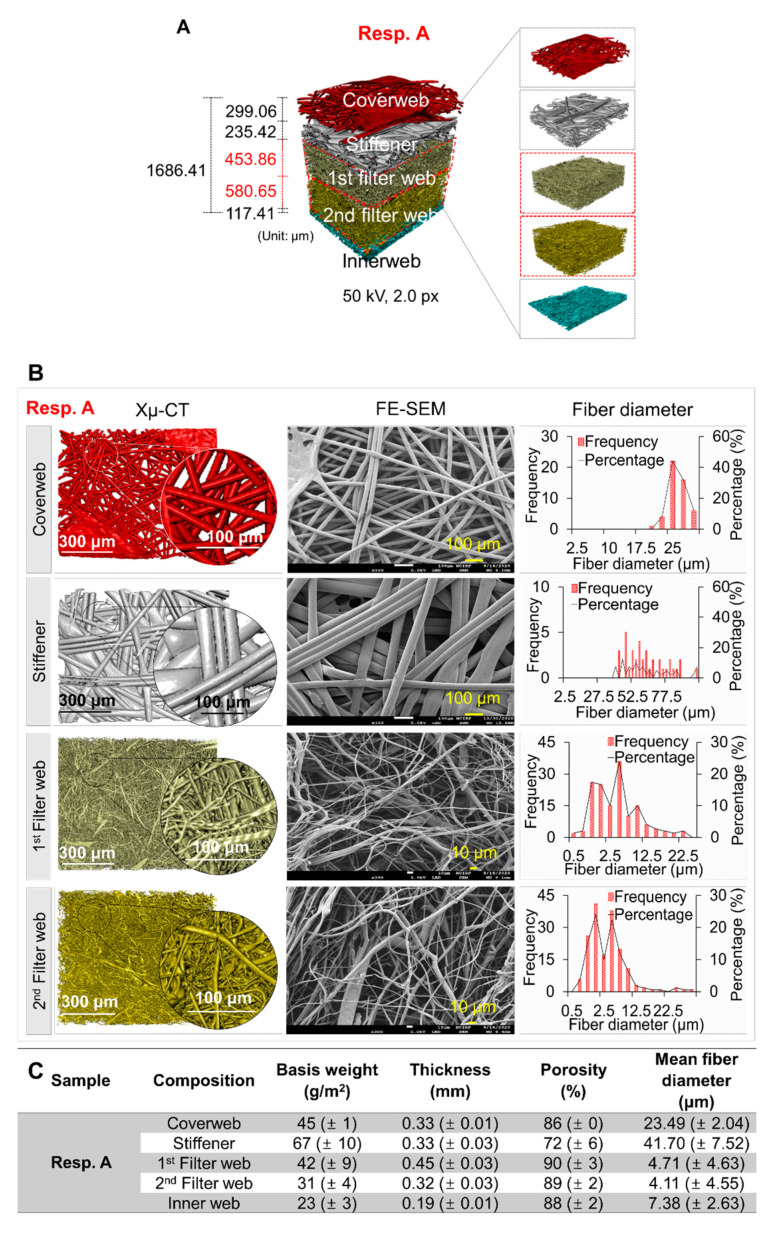

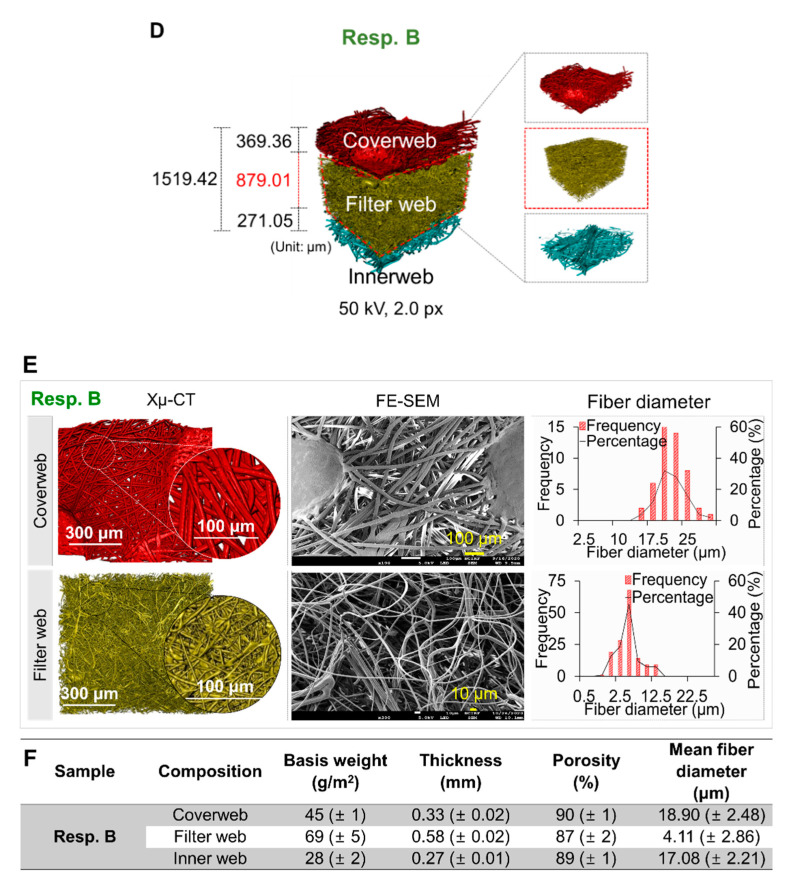
(**A**,**D**) Layer structures of respirators visualized by X-ray computed tomography (Xµ-CT), (**B**,**E**) 2D images and fiber diameter distribution using Xµ-CT and FE-SEM, and (**C**,**F**) morphological parameters of respirator components. The fiber diameter distribution is suggested in frequency and percentage, indicated as the red bar graph and black line, respectively.

The influence of various disinfection methods on the filtration and bactericidal performance was investigated for Resp. A and Resp. B. The 3D images of layer constructions for those respirators were analyzed by the X-ray computed tomography (Xμ-CT) (Figure 1). Common components of respirators included: a spunbond coverweb, one or two layers of electrostatically charged meltblown filter webs, and a spunbond inner web. respirator A (Resp. A) had an additional stiffener web and 2 layers of meltblown webs. The meltblown filter web was comprised of very thin fibers <2.5 μm in a considerable portion. In contrast, respirator B (Resp. B) consisted of a single layer filter web of which fiber diameter ranged from 2.5 μm to 12.5 μm. The thickness of each layer of the respirators was measured firsthand with a thickness gauge applying a pressure of 2.4 N, and is presented in Figure 1C,F. It is noted that the measurements by the gauge were smaller than those estimated from the specific locations of Xμ-CT images. When analyzing the morphological changes in the later sections, first-hand measurements were used.

### 3.1. Filtration Performance with Varied Disinfection Treatments

**Figure 2:**
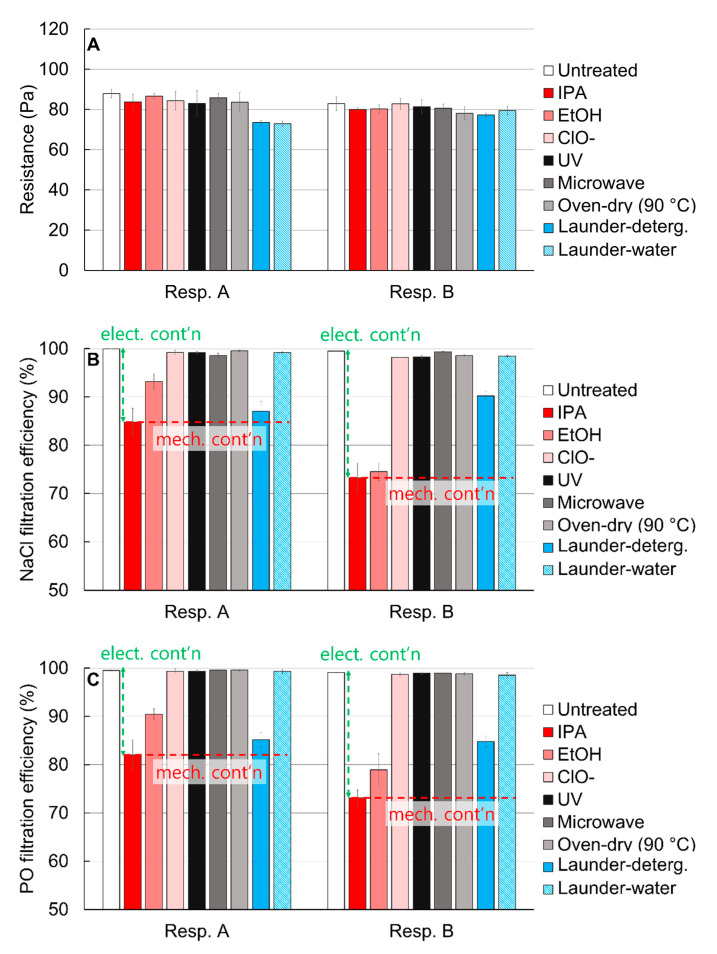
Filtration performance of respirators with varied disinfection treatments, corresponding to (**A**) resistance, filtration efficiency against (**B**) NaCl and (**C**) paraffin oil. The red dotted line indicates the presumed contribution of mechanical filtration, while the green dotted line indicates the contribution of electrostatic filtration.

Varied disinfection methods accessible to the general public were investigated for their effects on filtration performance. Without disinfection treatments, both respirators showed very high filtration efficiency of ≥99.5%. It should be noted that the filtration efficiency was measured after challenging the respective aerosols for 3 min (by the KF standard) [35], which corresponded to about 2.7 mg of NaCl and 8 mg of paraffin oil mass. The filtration performance against NaCl and paraffin oil aerosols were comparable, and the effects of various treatments on filtration of either aerosol were very similar (Figure 2).

The resistances of respirators were mostly consistent regardless of treatments, except that laundering treatments slightly decreased the resistance of Resp. A. The physical characteristics of respirators before and after treatment were further investigated in later sections. As for the filtration efficiency, the organic solvents such as IPA and EtOH, and detergent-laundering deteriorated the filtration efficiency of both respirators. Particularly for IPA and EtOH treatments, up to ~28% of efficiency was lost after treatments. Considering that the resistance of the solvent-treated samples was unchanged, it can be inferred that the solvents caused the reduction of filtration performance by affecting the electrostatic filtration capacity, rather than disrupting the structural integrity. Similarly, the filtration efficiency was notably reduced after laundering with detergent. As laundering with water barely affected the filtration efficiency, this indicates that the detergent negatively affected it, probably through an impact on the electrostatic capture mechanism.

Physical treatments employing UV irradiation, oven-dry, and microwave irradiation caused little impact on the filtration efficiency or resistance. Previous studies reported the deteriorated performance with thermal treatment of electret filters [10,41], but most of the performance deterioration occurred with harsher conditions (120 °C, 48 h) and especially for materials with high dielectric constants. Aging the electret filter at an extremely high temperature can trigger the mobility of polymer chains and charge carriers, leading to the loss of charges and increased particulate penetration [41]. In this study, the polypropylene electret filters were treated by the oven-dry condition that was relatively mild and short-term (90 °C, 1 h); and for this reason, the oven-dry treatment caused negligible effect on the performance change. UV irradiation may influence the performance by causing the surface oxidation, turning the surface hydrophilic [42]; and the increased conductivity in a humid condition can lead to the loss of charges, reducing the filtration efficiency [43,44]. However, UV irradiation, at the level of 10 W power with 253.7 nm wavelength, of this study did not significantly affect the filtration efficiency.

The results in Figure 2 show that thermal (oven-dry and microwave) and UV treatments hardly affected the filtration performance, aside from the fact that microwaving is not recommended for the safety reasons with metal components. Samples laundered with water maintained the performance as the untreated ones. However, the probable structural change, the torn coverweb (Figure S3), during the laundering procedure is of concern. Thickness and porosity of filter webs after treatments showed little differences compared to the untreated webs (Table S1). The residues on fibers after detergent-laundering is also of concern in the regard of environmental impacts (Figure S5).

### 3.2. Charge Decay

**Figure 3:**
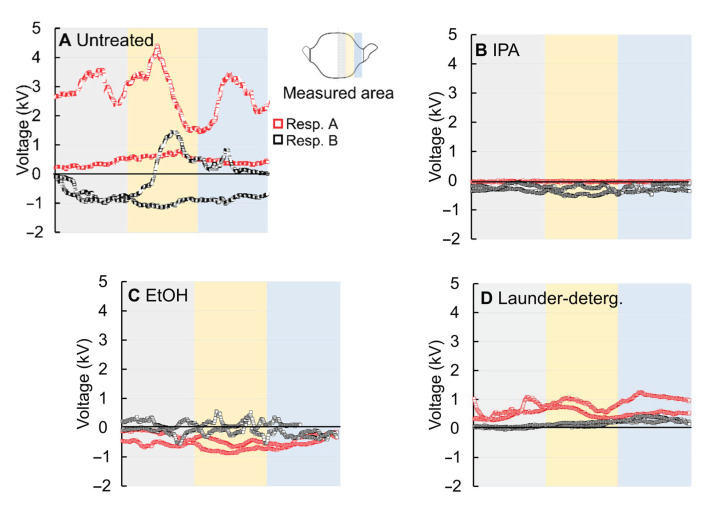
Surface potential of respirators with varied treatments, corresponding to (**A**) untreated, (**B**) IPA-immersed, (**C**) EtOH-immersed, and (**D**) laundered with detergent. The potential was measured by line-scanning in a horizontal direction across the shaded area.

To examine the cause of reduced performance, the surface potential of the filter web was measured (Figure 3 and Figure S6). The surface charges of the filter media are not consistently positive or negative; instead, both positive and negative charges can exist simultaneously, compensating the overall charges on the filter surface [45]. Therefore, for such cases, the average surface potential over an area can be less meaningful than the variation of potential. In Figure 3, the surface potential across the horizontal line was measured by line-scanning, and the fluctuation of the voltage values was observed as an important parameter. The surface potential of the untreated filter webs from respirators A and B ranged from −1.6 kV to +4.4 kV, and these surface charges contributed to particle capture either by coulombic attraction or induced polarization [46]. When the electret media was exposed to IPA, EtOH, and detergent solution, the range of surface potential was considerably reduced. In particular, IPA-treated respirator exhibited nearly 0 kV invariably across all areas, clearly indicating the loss of surface potential. The EtOH or detergent-treated respirators showed a slightly larger variation than the IPA-treated ones, indicating that surface charges may not be completely lost by those treatments. The remaining charges would contribute to electrostatic filtration mechanism, as indicated by the higher filtration efficiency of EtOH and detergent-treated respirators compared to that of the IPA-treated ones (Figure 2).

**Figure 4:**
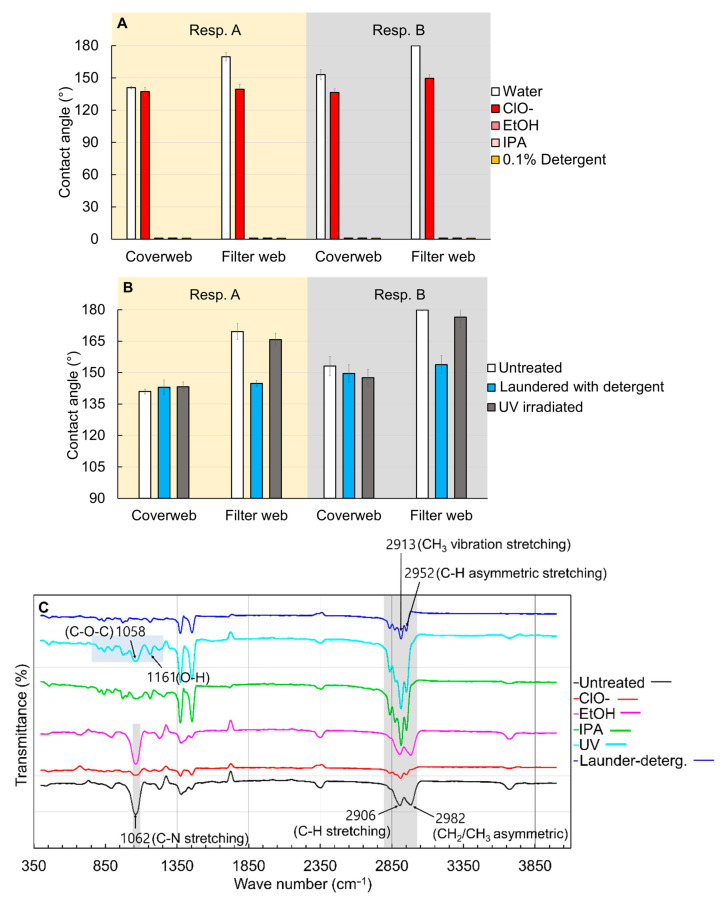
Wettability and chemistry of webs with different treatments. (**A**) Contact angle of various liquids on the coverweb and filter web, (**B**) water contact angle, and (**C**) FTIR-ATR transmittance of the differently-treated media. Contact angles were measured in 60 s upon placing the liquid drop. For EtOH, IPA, and detergent solution, the contact angles were 0°.

The charge decay occurred by the exposure to alcohol and detergent solution was probably due to the mobility of the charge carrier caused by the penetration of liquid [9,24]. Water has high surface tension and does not wet the polypropylene (PP) surface; therefore, the effect of water-immersion on PP media is negligible. IPA and EtOH immediately wetted the PP media in each layer as represented by the contact angle ~0° (Figure 4); and penetration of solvents into PP molecules expedited the charge carrier mobility, quickly dissipating the charges. In the case of 0.1% detergent solution, instantaneous contact angle was around 120°, then within 30~60 s, the PP web fully absorbed the liquid droplets. As the affinity of detergent solution to PP was not as high as EtOH or IPA, the impact of detergent on the electrostatic filtration was not as significant as that of organic solvents. Unlike the other test liquid, the ClO^−^ droplet maintained the contact angle of 140° without spreading, and the electrostatic filtration was hardly affected (Figure 2). Among the treatments, detergent treatment reduced the contact angle; this increased surface wettability of filter web would adversely influence the charge retention by increasing the electric conductivity, as evidenced from the surface potential measurement in Figure 3. As a result, the filtration performance after detergent treatment significantly decreased (≥10.8%).

To examine whether UV-treated or detergent-laundered respirators changed their chemistry, contact angles and FTIR spectra from those surfaces were examined (Figure 4B,C). Untreated PP media has C-H stretching (2906 cm^−1^), CH_2_/CH_3_ asymmetrical bending (around 2982 cm^−1^), and other carbon and hydrogen bonding (2835 to 2972 cm^−1^) peaks [47,48]. After UV irradiation, distinctive peaks were observed for: C-O-C (around 1058 cm^−1^) and O-H bending (around 1161 cm^−1^) [49,50]. The changed surface chemistry seemed to attribute to surface oxidation, but hardly affected the wettability and filtration performance after UV treatment. Solvent treatments with IPA, EtOH, and ClO^−^ showed neither noxious residues nor distinctive hydrophilic bonding.

### 3.3. Mechanical Filtration

The particle capture of an electret filter is contributed by both mechanical and electrostatic capture mechanisms. An electrostatic capture mechanism significantly improves the quality factor (filtration performance per a unit resistance); and when the surface charges are decayed, filtration mostly depends on the mechanical capture of particles. It is well accepted that IPA treatment removes the surface charges of filter media; therefore, the IPA-treated samples can be deemed as the mechanical filter (mechanical contribution noted as “mech. cont’n”) (Figure 2 and Figure 3B). The performance above this level of filtration for the untreated respirators A and B is considered as the electrostatic capture mechanism (electr. cont’n). Disinfection treatments such as solvent immersion, UV, oven-dry, and microwaving seemed to have negligible impacts on the structural integrity (Figures S3 and S4); accordingly, there were no apparent changes in the resistance and mechanical filtration after those treatments. Notable fiber damage was found in the laundered samples by the external force imposed during laundering (Figure S3A). The physical damage of Resp. A resulted in the decrease of resistance and mechanical filtration performance, corresponding to the lower filtration efficiency compared with Resp. B.

### 3.4. Bactericidal Performance of Disinfection Treatments

**Figure 5:**
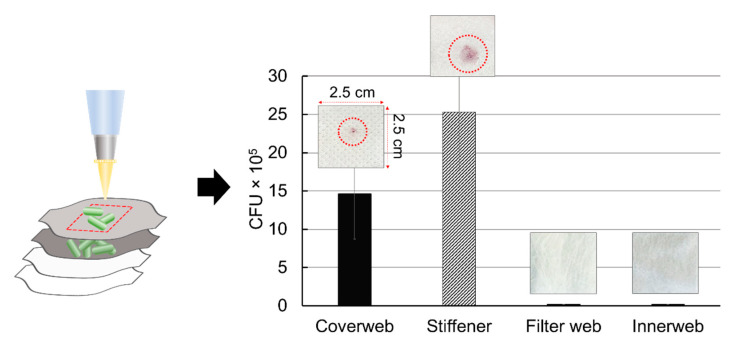
The CFU of loaded *E. coli* on each layer of untreated respirator A (Resp. A).

To investigate the disinfection efficacy, the number of survived colony on respirators after disinfection treatments was quantified. The *E. coli* culture was loaded on the respirators applying physical pressure; the bacteria culture penetrated up to the stiffener, and did not penetrate beyond this layer (Figure 5). Without the physical pressure, the loaded culture drop rolled around on the surface of coverweb. The number of loaded *E. coli* by each layer was correlated with OD_470_ measurement and expressed as CFU/cm^2^ substrate surface. Bacteria inactivation or removal efficiency was calculated by: $\frac{\left(B_{0}-B_{n}\right)}{B}\times 100\;\left(\%\right)$, where B_0_ is the number of live bacteria in the untreated sample area, and B_n_ is the number of live bacteria in the treated sample area (n = 1~8, each number indicating the eight disinfection methods).

**Figure 6:**
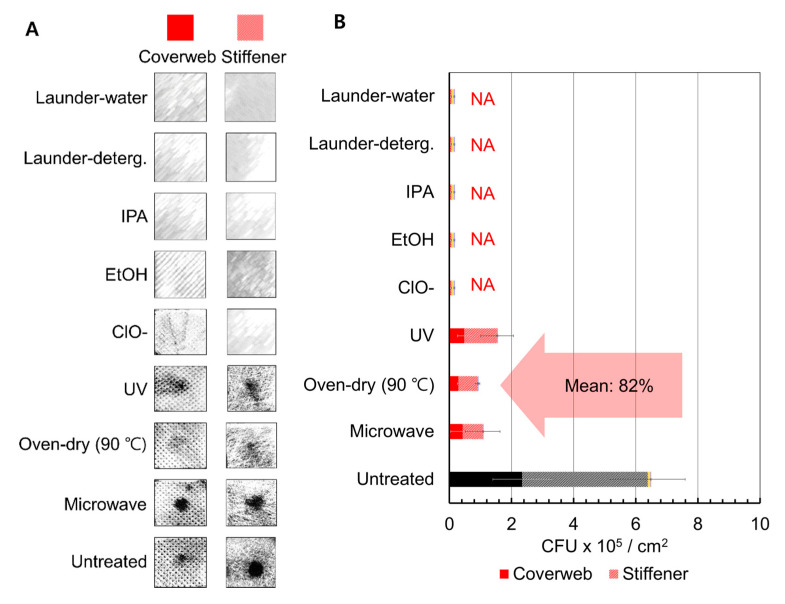

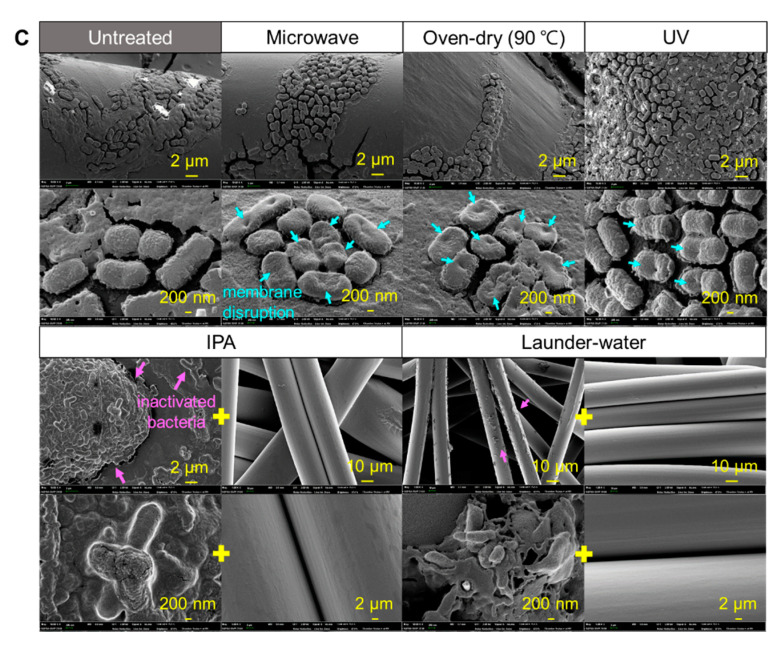
Numerical analysis of bacteria for each layer of Resp. A with varied treatments. (**A**) Monochrome calibrated images of coverweb and stiffener with bacteria cells, and (**B**) quantification of live *E. coli* remaining on each layer with and without disinfection treatments. As the bacteria was unable to penetrate through meltblown filter web and inner web, the images of only coverweb and stiffener are presented. (**C**) FE-SEM images of loaded bacteria after microwaving, oven-dry, UV irradiation, IPA immersion, and water-laundering.

Bactericidal or bacteria removal efficiency of Resp. A is shown in Figure 6 and Figure S7. In the 2.5 cm × 2.5 cm designated area of each web, non-treated samples showed ~1.46 × 10^6^ and ~2.53 × 10^6^ CFUs on the coverweb and stiffener, respectively. Microwaving, oven-dry, and UV treatment showed similar bactericidal effect up to about 82%; in fact, this is inconsistent with previous studies [18,20], where 99.99% inactivation efficiency was achieved. This discrepancy can be attributed to the different treatment conditions, such as irradiation intensity, treatment time, and amount of bacteria loaded. In a previous study, 1.0 × 10^6^ CFU of bacteria was loaded on the respirator with a nebulizer; and the UV irradiation (wavelength; 254 nm, 5 min) and microwaving (400 W, 10 min) resulted in complete inactivation of cells [18]. The case of an oven-dry treatment (100 °C, 15 min) also achieved 99.99% inactivation efficiency, when 5.0 × 10^4^ CFU of bacteria was dropped onto the surface of the respirator [20]. Compared to previous studies, this study built a harsher experimental condition with a higher loading of bacteria (5.0 × 10^6^ CFU). As a result, the intensity of microwaving (750 W, 2 min), UV irradiation (wavelength; 253.7 nm, 2 h), and oven-dry (90 °C, 1 h) of this study were insufficient for complete sterilization [51]. Chemical solvents (ClO^−^, EtOH, and IPA) and laundering treatments showed no color change with cell staining, indicating 100% bactericidal or bacteria removal efficiency (Figure 6 and Figure S7).

The morphological state of loaded bacteria after disinfection treatments was analyzed by FE-SEM (Figure 6C). With microwaving, oven-dry, and UV irradiation, disruption of *E. coli* membrane integrity was observed; on the contrary, IPA immersion and water-laundering resulted in both cell deformation and cell detachment. Remaining bacteria on water-laundered media was observed in a tangled structure of cells with contaminants on the fiber surface. Generally, microwaving and oven-drying rely solely on the heating effect to inactivate microorganisms [52], as heating triggers the denaturation of cell proteins [53]. Additionally, the UV irradiation breaks the strong bond of cell membranes, such as O-H, P-O, and N-H bonds of *E. coli*, or induces oxidation, leading to cell death [54]. The solvents and detergent laundering treatments have compound effect of bactericidal and bacteria removal activity, by accumulating in the lipid of membrane and degrading the membrane integrity while inducing suspension of bacterial droplet into the liquid [55,56]. Meanwhile, laundering effectively removed bacteria by detaching them from fibers, rather than sterilizing the cells.

The results of bactericidal performance test imply the chemical solvents and hypochlorite solution are effective disinfection agents, as they concurrently inactivate the cell and detach bacteria. Even if it is not complete sterilization, UV irradiation, and oven-dry methods seem fairly applicable for inactivating cells to some extent. Laundering treatment removed a considerable amount of loaded bacteria; additionally, even the remaining bacteria was inactivated.

## 4. Conclusions

For the environmental protection during a global pandemic under the supply shortage, the reusability of disposable respirators was inevitable. This study evaluated the effects of varied disinfection treatments of disposable respirators on the filtration performance, morphological integrity, and bactericidal effectiveness, adopting eight different disinfection methods. It was confirmed that disinfection methods including laundering and chemical treatments effectively reduced the risk of bacterial infection via inactivating or removing adhered bacteria; however, they significantly deteriorated the filtration performance, dissipating the electrostatic force of the fibers. The oven-dry and UV irradiation maintained the performance, but showed incomplete sterilization in a harsh microbial environment. The results of this study will ultimately contribute to the advancement of environmental health and safety by providing a scientific background on the effect of disinfection treatment methods. Further investigation simulating the actual pandemic environment is recommended to identify the effective methods in the varied infectious conditions. Additionally, a potential risk of secondary infection by the released active bacteria back to the environment needs to be further investigated, to suggest the most relevant disinfection method for public health and environment. The scope of this study remains in bacteria disinfection. Further study with virus is recommended.

## Figures and Tables

**Table 1:** Summary of previous studies examining the disinfection methods for respirators.

Disinfection Method	Filtration Performance	Bactericidal/Virucidal Activity	Reference
Oven-dry	No drop in filtration efficiency under 75~100 °C for 30 min treatment; sharp drop in filtration efficiency when treated with 125 °C (up to 10%)	About 5-log_10_ fold reduction of SARS-CoV-2 under 95 °C for 5 min treatment	[7]
	No drop in particle filtration efficiency under 100 °C, 50 min treatment	Above 4-log_10_ attenuation in Tulane virus, rotavirus, adenovirus	[8]
	Small decrease in particle filtration efficiency under 100 °C for 15 min (about 1.5%)	Complete bactericidal efficiency of *S. aureus*	[20]
Microwaving	No particle filtration efficiency drop for 2 min exposure	NA	[10]
	No particle filtration efficiency drop for 2.5 min exposure	>4-log_10_ reduction of *E. coli* for 2.5 min exposure with 500 W power	[18]
UV irradiation	NA	Complete bactericidal efficiency to *B. subtilis* spores	[12]
	No particle filtration efficiency drop	NA	[7,10,12,17]
		Complete bactericidal efficiency of *S. subtilis* and *E. coli*	[18]
	Small decrease in particle filtration efficiency (up to 1.25%)	NA	[16]
Chlorinated disinfectant	No drop in particle filtration efficiency after using hypochlorite wipe	About 1-log_10_ attenuation in *S. aureus* after using hypochlorite wipe	[13]
	Decrease in particle filtration with increased pressure drop (up to 18.3%)	NA	[11]
	No effect on aerosol penetration efficiency	NA	[10,14]
Ethanol	Drastic decrease in filtration efficiency (up to 18%)	Complete suppression of bacterial growth after 5 min immersion	[22]
		NA	[7,19]
		Complete bactericidal efficiency of *S. subtilis* and *E. coli*	[18]
Isopropanol	Decrease in particle filtration efficiency (over 5%)	NA	[11,17]
	Significant drop in oily aerosol filtration efficiency after 2 min immersion (up to around 40%)	NA	[9]
Detergent -laundering	Significant drop in particle filtration efficiency after soap solution soaking for 2 min (up to around 39%)	NA	[15]

## Data Availability

The data presented in this study are available on request from the corresponding author.

## Supplementary Materials

The following are available online at https://www.mdpi.com/2073-4360/13/1/45/s1, Section S1–S5: Detailed methods and information; Figure S1. The schematic diagram of the image visualization using Xμ-CT; Figure S2. The procedure of bactericidal/bacteria removal efficiency test; Figure S3. Microscopic and FE-SEM images of filter webs from respirators after various treatments; Figure S4. Xµ-CT images of IPA-immersed and detergent-laundered filter webs; Figure S5. Energy dispersive spectroscopy (EDS) and elemental mapping for treated filter webs; Figure S6. Surface potential measurements of treated filter webs; Figure S7. %Bactericidal or bacteria removal efficiency of varied disinfection treatments; Table S1. Morphological characteristics of filter webs with varied disinfection treatments.
