# Supplementary material for: Disinfection Treatments of Disposable Respirators Influencing the Bactericidal/Bacteria Removal Efficiency, Filtration Performance, and Structural Integrity
Source: Polymers (Basel). 2020 Dec 24;13(1):45. doi: 10.3390/polym13010045 (PMC7796291; doi:10.3390/polym13010045)
Supplement: Supplementary file 1 [file polymers-13-00045-s001.pdf]

polymers

MDPI

Supplementary Materials: Disinfection Treatments of Disposable Respirators Influencing the Bactericidal/Bacteria Removal Efficiency, Filtration Performance, and Structural Integrity

Seojin Jung, Tahmineh Hemmatian, Eugene Song, Kyeongeun Lee, Dongwan Seo, Jehyung Yi, and Jooyoun Kim

Section S1: Xμ-CT analysis

To visualize respirators in 3D image using X-ray computed tomography (Xμ-CT), approximately 1000 slices representing a 2D image were taken, and reconstructed into a 3D image. After generation of 3D structure, further characterization of Xμ-CT image was performed using Dragonfly Pro software (Object Research Systems, Montréal, Qc, Canada). The multiple layers were separated for the coverweb, stiffener, filter web, and inner web based on the fiber characteristics and gap between the layers. The Xμ-CT enables observing the internal structure of each layer in all directions.

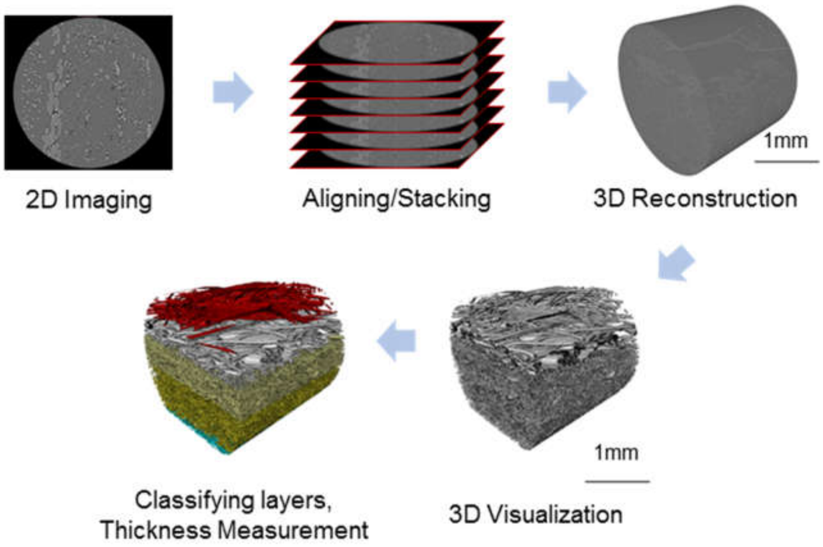

Figure S1. The schematic diagram of the image visualization using X-ray computed tomography (Xμ-CT).

Section S2: Quantification of bacteria on the web

Figure S2 presents the procedure of quantifying the bactericidal/bacterial removal performance by the staining-colorimetric method. With the colorimetric method, the bacterial inactivation effect can be observed with bare eyes directly without having to detach the cells from fibers. For quantification, the bacteria-loaded area was cut into 2.5 cm × 2.5 cm, and every and each layer of the respirator material was analyzed separately, staining with 200 μL of 0.25 % (w/v) iodonitrotetrazolium (INT) (Sigma-Aldrich, USA) dissolved in the phosphate-buffered saline (pH 7.4 PBS, Thermo Fisher Scientific, USA). The INT-immersed samples were incubated at 37 °C for 4 h, where INT stained the live cells in purple, forming a formazan. After incubation with INT, the formazans formed on samples were extracted with 1 mL of dimethyl sulfoxide (DMSO, Daihan Scientific, Korea). The extract was then filtered through a microfilter (0.22 μm pore), and the formazan eluent was measured for its absorbency at the wavelength of 470 nm (OD_{470}) using a spectrophotometer (SpectraMax 190, Molecular Devices LLC, USA). The CFUs of cells were estimated by using a standard curve and converted to CFU/cm^2 substrate surface.

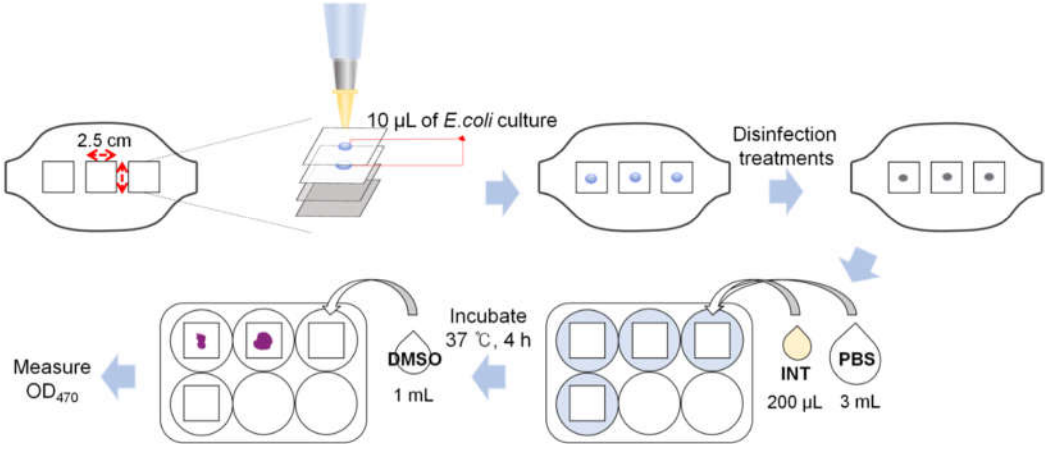

Figure S2. The procedure of bactericidal/bacteria removal efficiency test.

Section S3: Morphological characteristics of filter webs

The physical integrity of filter webs after disinfection treatments was investigated. From FE-SEM images, no valid structural changes such as swelling or damage were found in either type of respirator treated with chemical solvents or thermal, ultraviolet (UV) treatment (Figure S3), which corresponded with the unchanged resistance values (Figure S2). Distinctive fiber breakage or swelling was also not found for filter webs of both respirators after laundering; however, respirator A (Resp. A) is assumed to be more vulnerable to mechanical damage than respirator B (Resp. B), as it was composed of thinner fibers (Figure S1, fiber size distribution). While the reduced extent of electrostatic filtration would be similar for both respirator types, the reduction in mechanical filtration was larger for Resp. A, due to the physical damage. The thickness and porosity of filter webs after disinfection treatments showed no considerable difference compared with untreated webs (Table S1). From Xμ-CT images (Figure S4), the filter web laundered with detergent showed large variations (~ 10%) of thicknesses depending on the measurement locations, probably due to the local disintegration of fibrous assembly.

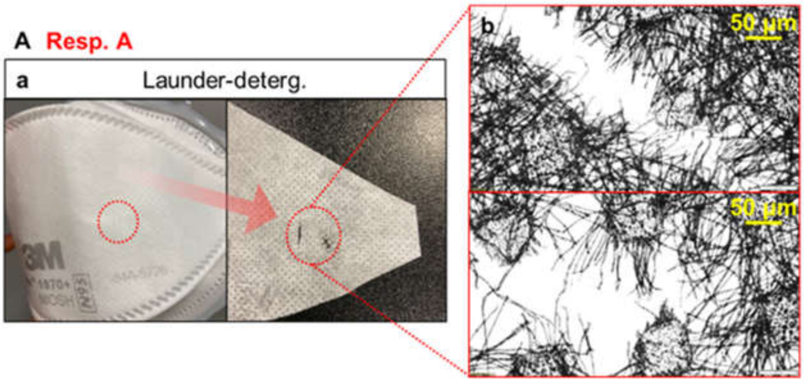

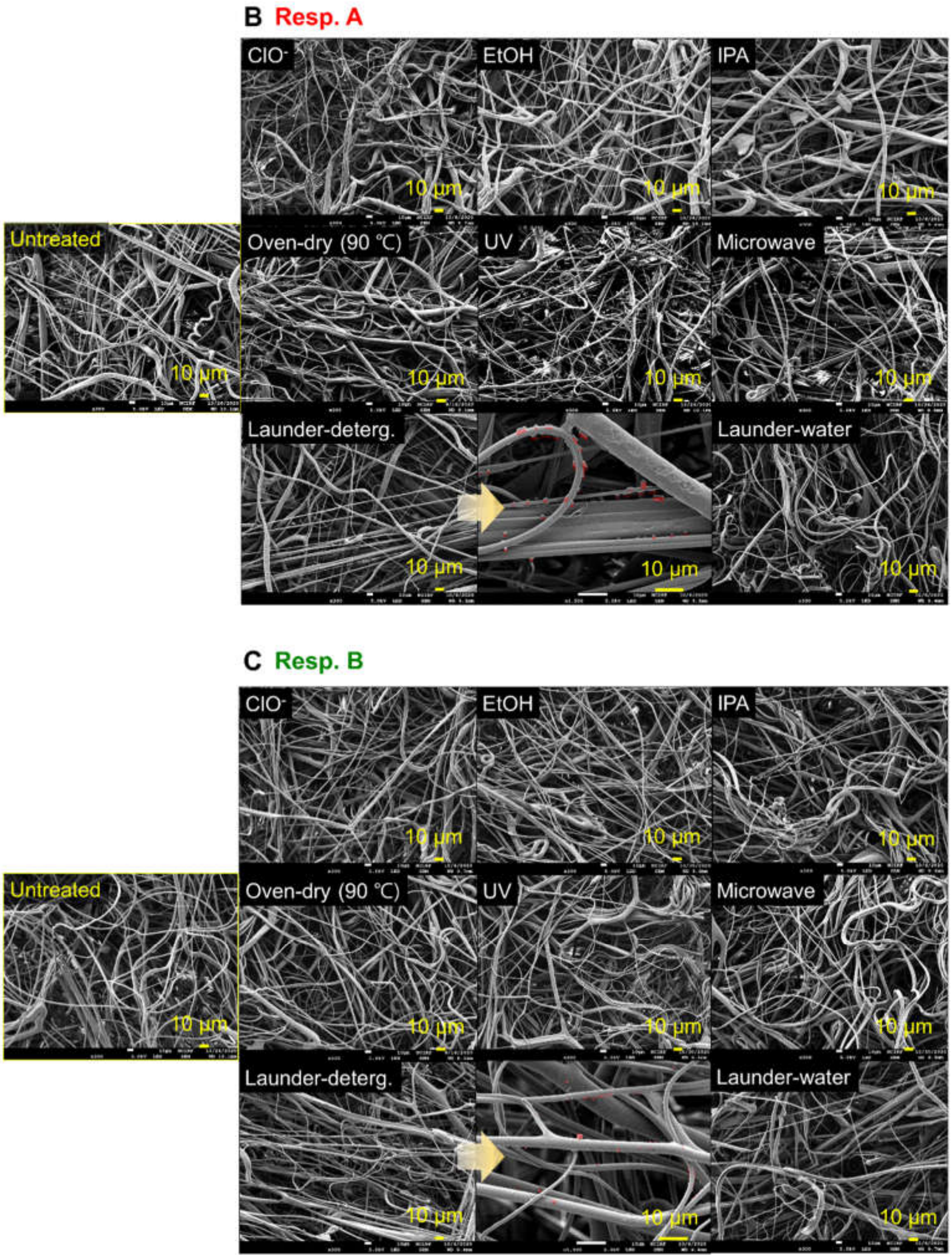

Figure S3. (**A**) Discernible structural damage of respirator A (Resp. A) after detergent laundering; (**a**) and (**b**) for the torn webs and breakage of fibers of Resp. A. There was no discernible deformation of respirator B (Resp. B). (**B**, **C**) SEM images of filter webs from Resp. A and Resp. B, respectively, after various treatments. Particles adhered to fibers after detergent-laundering are calibrated into red color.

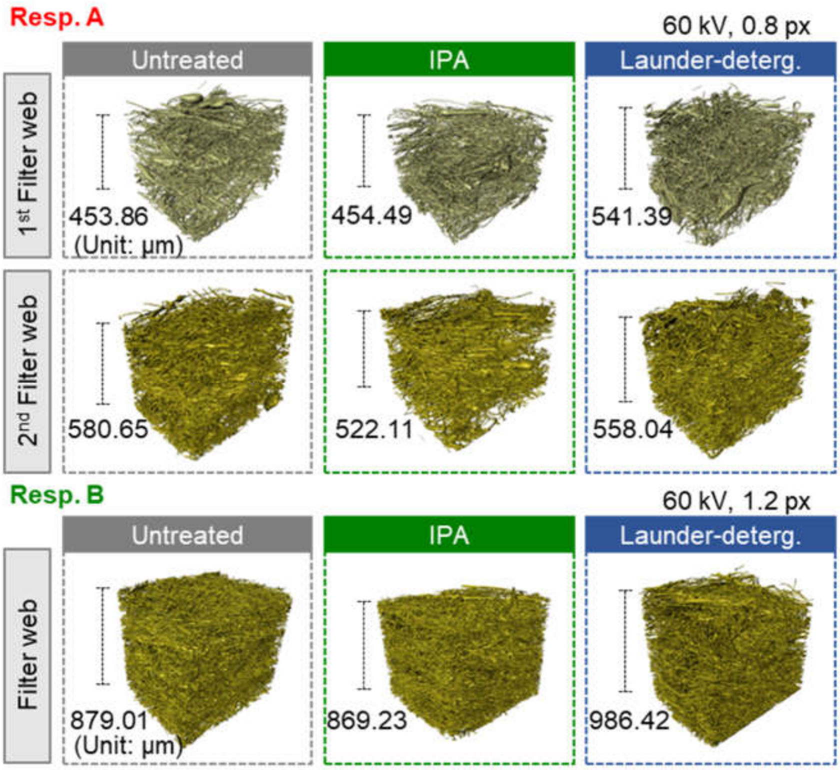

Figure S4. Xμ-CT images of isopropanol (IPA)-immersed and detergent-laundered filter webs.

Table S1. Morphological characteristics of filter webs with varied disinfection treatments.

**Treatment**	**Untreated**		**IPA**		**EtOH**	
**Property**	**Thickness (mm)**	**Porosity (%)**	**Thickness (mm)**	**Porosity (%)**	**Thickness (mm)**	**Porosity (%)**
Resp. A (2L filter)	0.75	86	0.77	87	0.70	85
Resp. B (1L filter)	0.60	89	0.59	87	0.70	87
Treatment	**ClO^-**		**UV**		**Microwave**	
Property	Thickness (mm)	Porosity (%)	Thickness (mm)	Porosity (%)	Thickness (mm)	Porosity (%)
Resp. A (2L filter)	0.70	86	0.76	87	0.79	88
Resp. B (1L filter)	0.68	89	0.63	88	0.62	89
Treatment	**Oven-dry**		**Launder-detergent**		**Launder-water**	
Property	Thickness (mm)	Porosity (%)	Thickness (mm)	Porosity (%)	Thickness (mm)	Porosity (%)
Resp. A (2L filter)	0.75	87	0.76	89	0.62	90
Resp. B (1L filter)	0.62	87	0.56	89	0.57	89

After laundering with detergent, Resp. A and Resp. B showed particles of 0.56 ~ 0.80 μm (marked in red color Figure S3), which consisted of carbon, oxygen and calcium from the EDS analysis (Figure S5). The calcium component is presumed to come from the reaction of tetrasodium ethylene-diamine tetraacetate, a light water softener component, with Ca^{2+} ion in tap water.

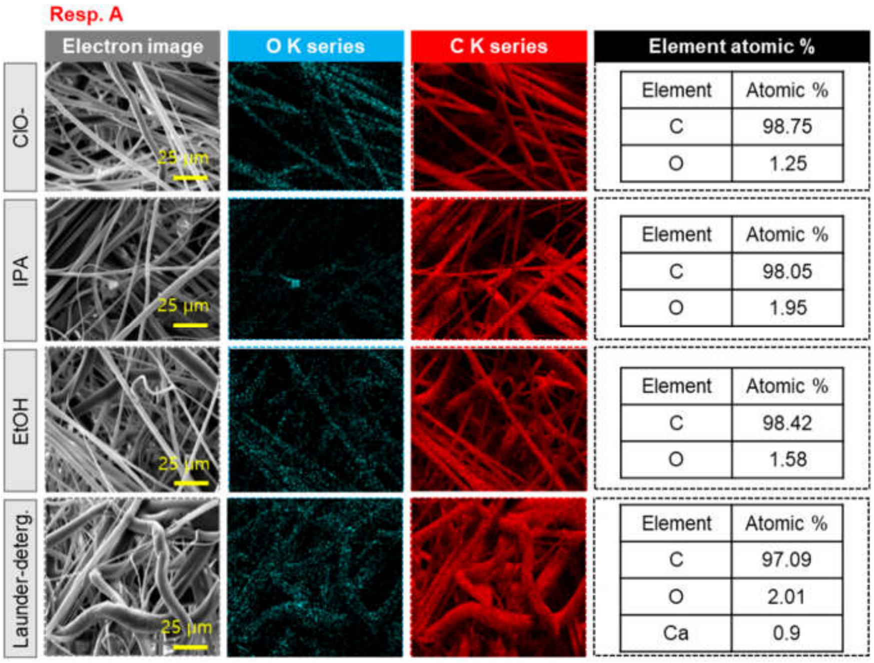

Figure S5. Energy dispersive spectroscopy (EDS) and elemental mapping for treated filter webs.

Section S4: Surface potential measurements

Surface potential measurements of treated filter webs, except for isopropanol (IPA)-immersed, EtOH-immersed, and laundered with detergent, are shown in Figure S6. Other than the alcohol and detergent treatments that significantly deteriorated the surface potential (Figure S3), filter webs mostly maintained the surface potential after treatments of UV irradiation, microwaving, oven-dry, ClO and water-laundering. The retained surface charges corresponded to the maintained particle filtration performance (Figure S2).

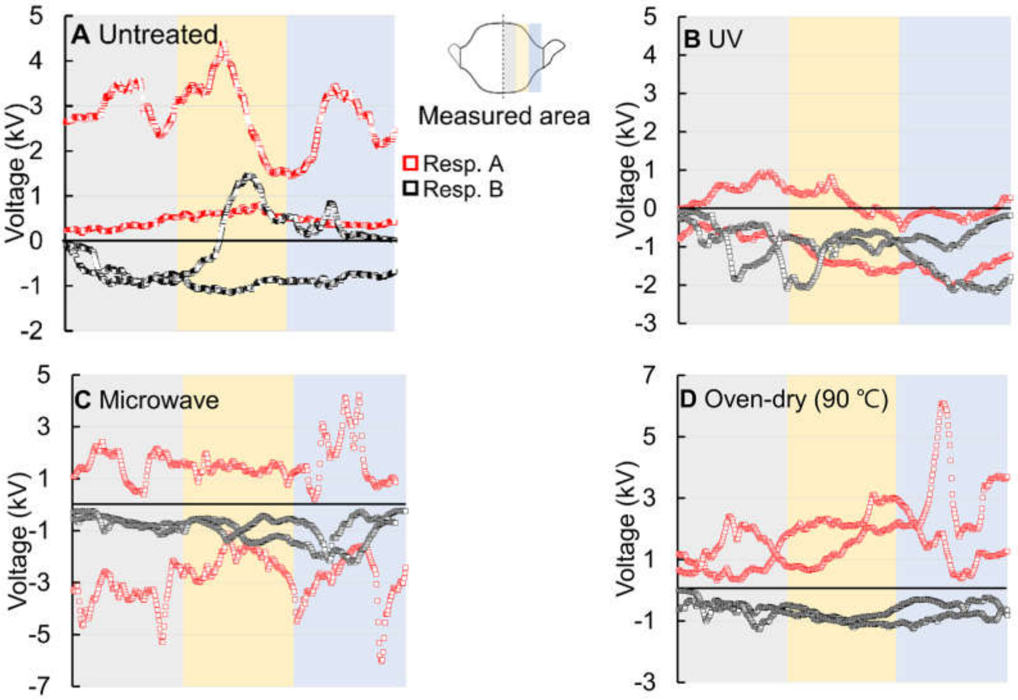

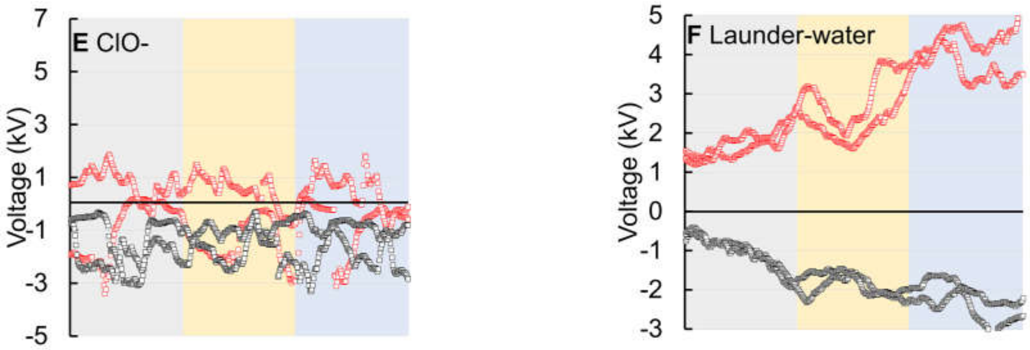

Figure S6. Surface potential measurements of treated filter webs, corresponding to (A) untreated, (B) UV irradiated, (C) Microwaved, (D) oven-dried, (E) ClO^--immersed, and (F) laundered with water.

Section S5: Bactericidal performance test

To evaluate the bactericidal or bacteria removal performance of disinfection treatments, *E. coli* culture was loaded on the respirators using a micropipette. In this process, the physical force was applied, so the bacteria culture can be loaded through the outer coverweb. When the cell culture was applied to Resp. A, the culture broth entered into the coverweb and stiffener, and did not penetrate beyond those layers. Among the treatments, UV irradiation showed the least bactericidal efficiency (Figure S7), probably due to the limited UV absorption through the layers.

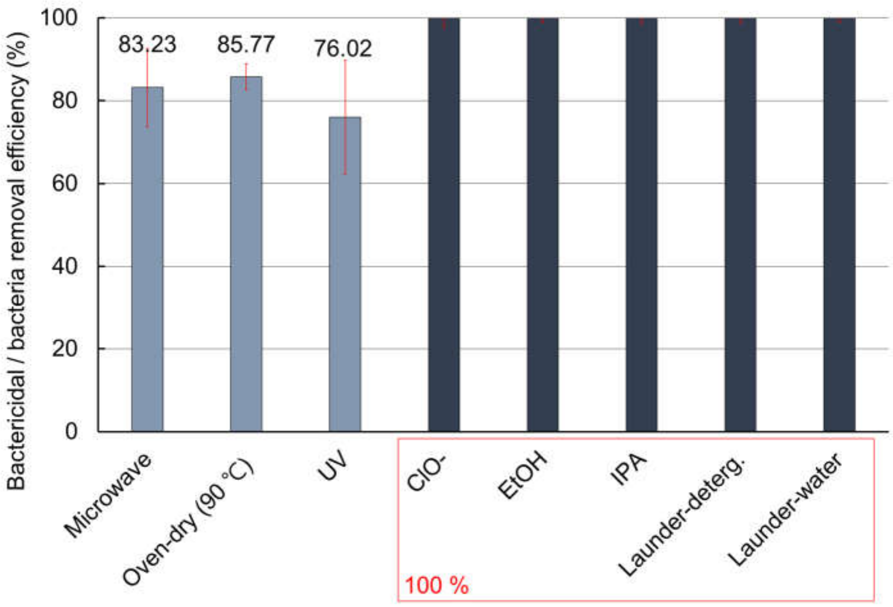

Figure S7. % Bactericidal or bacteria removal efficiency of varied disinfection treatments.

References

1. Garum, M.; Glover, P. W. J.; Lorinczi, P.; Drummond-Brydson, R.; Hassanpour, A. Micro- and Nano-Scale Pore Structure in Gas Shale Using Xμ-CT and FIB-SEM Techniques. *Energy Fuels* **2020**, *34*, 12340–12353.
2. Hemmatian, T.; Kim, J. Quantification Methods for Textile-Adhered Bacteria: Extraction, Colorimetric, and Microscopic Analysis. *Polymers* **2019**, *11*, 1666.
3. Wagner-Graham, M.A.; Barndt, H.; Sunderland, M.A. Measurement of Antibacterial Properties of Foil-Backed Electrospun Nanofibers. *Fash. Text.* **2019**, *6*, 1–11.
4. Qualls, R. G.; Johnson, J.D. Bioassay and Dose Measurement in UV Disinfection. *Appl. Environ. Microbiol.* **1983**, *45*, 872–877.
